# Rab GTPases in Immunity and Inflammation

**DOI:** 10.3389/fcimb.2017.00435

**Published:** 2017-09-29

**Authors:** Akriti Prashar, Laura Schnettger, Elliott M. Bernard, Maximiliano G. Gutierrez

**Affiliations:** Host-Pathogen Interactions in Tuberculosis Laboratory, Francis Crick Institute, London, United Kingdom

**Keywords:** Rab GTPase, macrophages, phagosomes, inflammation, innate immunity

## Abstract

Strict spatiotemporal control of trafficking events between organelles is critical for maintaining homeostasis and directing cellular responses. This regulation is particularly important in immune cells for mounting specialized immune defenses. By controlling the formation, transport and fusion of intracellular organelles, Rab GTPases serve as master regulators of membrane trafficking. In this review, we discuss the cellular and molecular mechanisms by which Rab GTPases regulate immunity and inflammation.

## Introduction

Eukaryotic cells have membrane bound organelles that are essential for maintaining cellular organization and performing highly dynamic and specialized functions. These processes, which depend on the transfer and exchange of cargo between different organelles, require communication within cells and between cells and their environment, while maintaining the distinct identities of these compartments. Regulated transport and trafficking of intracellular vesicles is required to achieve these highly coordinated and spatiotemporally regulated events (for a comprehensive review see reference Stenmark, 2009). In this context, intracellular trafficking and the immune function of cells are linked in multiple ways and this coordination is critical for dynamic and specialized immune defenses (Pei et al., 2012).

Firstly, intracellular trafficking regulates dynamic signaling-dependent immune responses. During microbial infections, pathogen recognition by specific receptors leads to signaling events that trigger appropriate immune responses. Interestingly, the activation of receptors by microbial ligands can result in completely different responses depending on the localisation of these receptors. The best characterized group of receptors that control dynamic signaling is the Toll-like receptors (TLR). Activation of these pattern recognition receptors on the plasma membrane leads to different signals than when the receptors are activated by microbial components localized in the lumen of endocytic vesicles (Gay et al., 2014).

Secondly, innate immunity is driven in specific cell types by different intracellular pathways, including the uptake of macromolecules, apoptotic cells, pathogens and pathogen derived vesicles. The general “inbound” trafficking of macromolecules into plasma membrane-derived vesicles occurs via the endocytic pathway (Conner and Schmid, 2003). Depending on the mechanism of uptake and the cargo being internalized, endocytosis is broadly categorized as phagocytosis, which is restricted to certain cell types, or as pinocytosis, which is performed by all cell types (Conner and Schmid, 2003; Huotari and Helenius, 2011).

Thirdly, lysosome-mediated microbial degradation is required for the activation of the antigen-specific adaptive immune responses, which provides long-lasting immunity (for review, see reference Iwasaki and Medzhitov, 2015). The processing of antigens by immune cells relies on the endocytic and phagocytic pathways, where antigens are degraded and loaded on receptors that are then exposed on the cellular surface. Once internalized, endolysosomal trafficking contributes to the degradation of the cargo for antigen presentation, which then primes the adaptive immune responses (Vyas et al., 2008).

Finally, along with mechanisms for internalizing cargo, cells have constitutive and regulated exocytic pathways responsible for secreting molecules, including cytokines, hormones and neurotransmitters (Gundelfinger et al., 2003). As the regulated secretion of cytokines and immune mediators is critical during immunity and inflammation, this cellular pathway plays a critical role in these processes.

**Figure 1:**
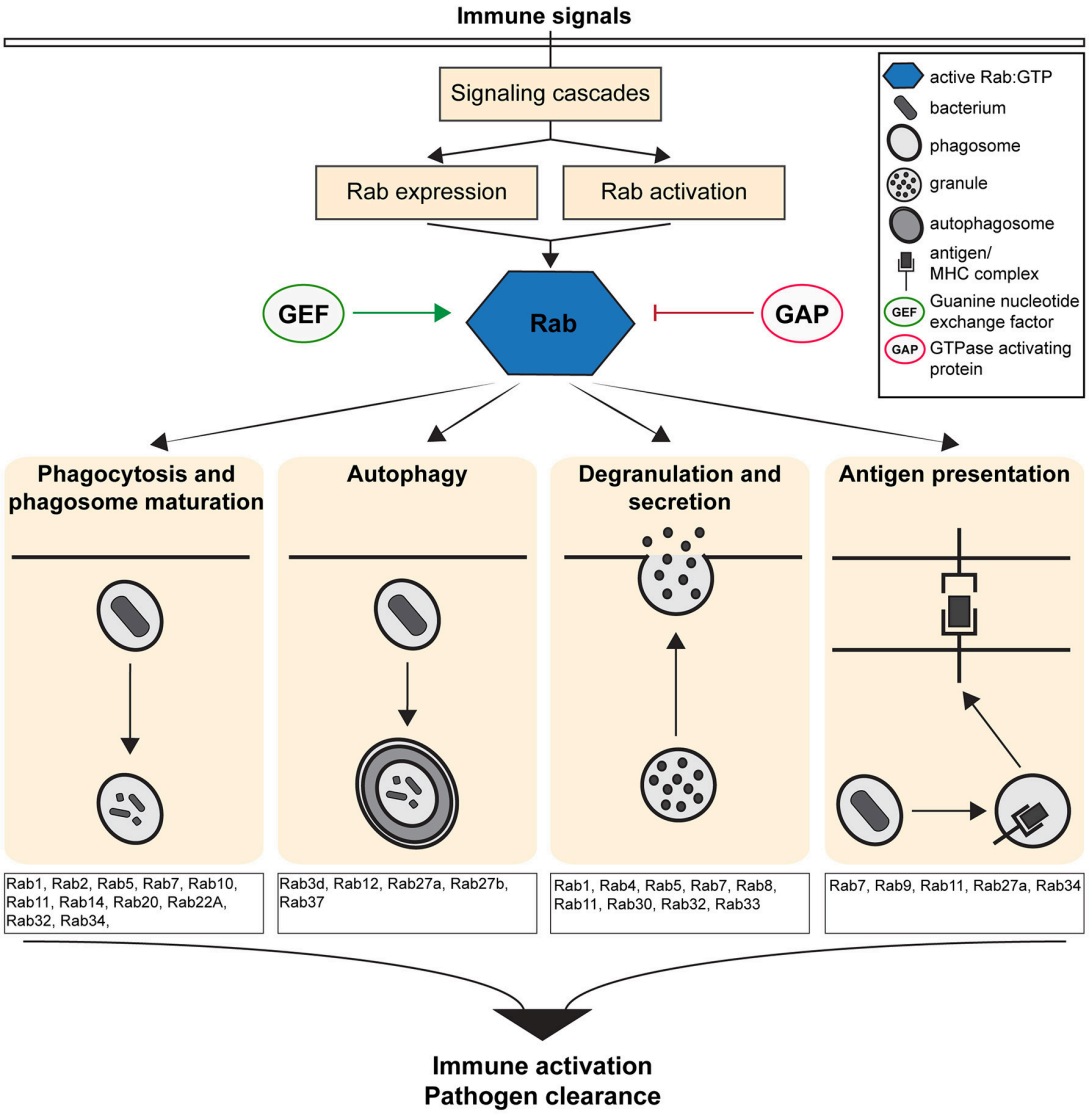
Overview of the Immune defense pathways regulated by the activity of Rab GTPases. Figure shows the cellular and molecular mechanisms by which Rab GTPases regulate immunity and inflammation by controlling the formation, transport and fusion of intracellular organelles.

The Rab (Ras related in brain) family of small GTPases regulates vesicular transport and determines organelle identities, thereby functioning as central players in regulating the intracellular and cell-cell communication required to generate and maintain cellular homeostasis (Zerial and McBride, 2001; Stenmark, 2009). Rab GTPases act as molecular switches that localize to distinct intracellular membranes and regulate intracellular trafficking at the level of vesicle budding, motility, tethering, docking and fusion through their interactions with specific effectors (Zerial and McBride, 2001; Stenmark, 2009). Therefore, Rab proteins act as scaffolds that integrate signaling and trafficking events, providing spatio-temporal control of organelle maintenance and trafficking (Schwartz et al., 2007). Given their critical role in regulating intracellular trafficking, Rab GTPases modulate immune responses by regulating the transport of immune receptors (Husebye et al., 2010), the secretion of chemokines and cytokines (Murray et al., 2005) and by up-regulating the critical immune surveillance processes of endocytosis and phagocytosis (Stenmark, 2009; Flannagan et al., 2012; Figure 1).

## Rab GTPases in innate immunity

Conserved microbe associated signatures, collectively referred to as pathogen-associated molecular patterns (PAMPs) are recognized by pattern recognition receptors (PRRs) on the surface of immune cells. This recognition induces intracellular signaling pathways responsible for inflammatory immune responses (Iwasaki and Medzhitov, 2015). In order to mount appropriate responses while avoiding chronic inflammation, intracellular trafficking must be tightly regulated in immune cells. Indeed, regulation occurs at least at two levels, including trafficking of PRRs and secretion of immune modulators (Schwartz et al., 2007). Phagocytes, namely macrophages, dendritic cells (DCs) and neutrophils, are critical components that drive the innate immune response. These cells engulf and destroy invading pathogens and drive customized adaptive immune responses (Iwasaki and Medzhitov, 2015). This process of pathogen uptake and destruction requires the concerted efforts of several members of the Rab family of small GTPases (Stenmark, 2009).

### Phagocytosis and phagosome maturation

Phagocytosis is the most important pathway implicated in the clearance of dying cells and microbial pathogens and hence plays a central role in tissue remodeling and immunity (Flannagan et al., 2012). After internalization of microbes, the initially formed nascent phagosome acquires the microbicidal and degradative properties necessary for pathogen clearance during a process called phagosome maturation (Flannagan et al., 2012). Hereby, the sequence of fusion with compartments of the endocytic pathway, as well as recycling of components from the phagosome is essential and highly regulated by Rab GTPases (Gutierrez, 2013). According to different proteomic studies performed in different model systems, at least 20 Rab GTPases are dynamically associated with phagosomes. However, the function of many of these Rab GTPases during phagosome maturation is still not well-characterized (Gutierrez, 2013). Rab5, together with Rab7, is one of the best-characterized Rab proteins in both endocytosis and phagocytosis (Vieira et al., 2003). Rab5, Rab22A and Rab14 are among the Rab GTPases present on early phagosomes where they regulate fusion with early endosomes that is required for the progression of phagosome maturation (Gutierrez, 2013). Late phagosomes are predominantly associated with Rab7 and Rab34, which regulate their fusion with late endocytic compartments via distinct mechanisms (Harrison et al., 2003; Vieira et al., 2003; Seto et al., 2011; Kasmapour et al., 2012, 2013). In addition to the fusion with specific endocytic compartments, Rab11 and Rab10 regulate phagosomal recycling and Rab1 and Rab2 regulate the interaction of phagosomes with the endoplasmic reticulum (ER), post-Golgi and ER-Golgi intermediate compartment (ERGIC) (Gutierrez, 2013). The interferon-γ (IFN-γ) inducible GTPase Rab20 is also present on phagosomes and links immune activation by this cytokine with phagosome maturation (Trost et al., 2009; Pei et al., 2014). Rab32, which is involved in the trafficking of lysosome-like compartments, the lysosome related organelles (LROs), which include melanosomes, lytic granules and neutrophil granules (Dell'Angelica et al., 2000), also associates with latex bead phagosomes and is implicated in the acquisition of the lysosomal enzyme cathepsin D by phagosomes (Seto et al., 2011; Gutierrez, 2013). However, precisely how all these Rab proteins orchestrate the interactions with specific subsets of early and late endosomes in time and space is less clear. It is also unclear if there are significant levels of redundancy in the pathway, since many of the phagosomal Rab GTPases seem to regulate fusion with late endocytic organelles in general. More importantly, the role of most of these GTPases in pathogen control by immune cells is still poorly defined.

Similarly to phagosome maturation, macropinosome formation is also regulated by Rab GTPases (Egami et al., 2014). In this process, Rab5 and Rab34 are required for the formation of actin-rich membrane ruffles and macropinosomes (Sun et al., 2003; Porat-Shliom et al., 2008). The late endosomal and phagosomal Rab7 regulates fusion of macropinosomes with lysosomes (Racoosin and Swanson, 1993). Furthermore, Rab20 and Rab21 are also localized to macropinosomes although the precise function of these Rab GTPases on macropinosomes is not clear (Egami and Araki, 2012a,b).

Several pathogens are known to subvert host cell trafficking pathways by targeting Rab GTPases, altogether highlighting a crucial role of Rab-dependent trafficking in immunity (Brumell and Scidmore, 2007; Sherwood and Roy, 2013). For instance, *Legionella pneumophila* recruits Rab1 to the *Legionella-*containing vacuole to generate an ER-like compartment favorable for bacterial replication (Kagan et al., 2004). Early endosome localized Rab14 is critical for maintaining the phagosome maturation arrest of mycobacteria containing phagosomes (Kyei et al., 2006). In contrast, loss of Rab14 inhibits *Salmonella typhimurium* replication, likely by promoting the maturation and acidification of *Salmonella* containing phagosomes (Kuijl et al., 2013). Recently, Rab11 has been shown to play a role in the rupture of *Shigella* containing vacuoles, which is necessary for bacterial replication and cell-to-cell spreading (Mellouk et al., 2014). When overexpressed as a GFP fusion protein, Rab32 is recruited to phagosomes containing *Mycobacterium tuberculosis*, as well as *Staphylococcus aureus* where it regulates the recruitment of the lysosomal enzyme cathepsin D (Seto et al., 2011). However, the specific function and involvement of Rab32 in the restriction of mycobacterial replication remains to be established. Interestingly, *S. typhimurium* was shown to interfere with the recruitment of Rab32/Rab38 and Rab29 (Rab7L1) to its vacuole, events not associated with the human-restricted *S. typhi*. These observations imply that some Rab GTPases can contribute to host specificity (Spano and Galan, 2012; Spano, 2016; Spano et al., 2016).

### Degranulation, secretory granules, and exocytosis

During the development of the immune response and inflammation, DCs, neutrophils and tissue resident macrophages produce immune mediators that are crucial for the resolution of inflammation and protecting the body against infection and injury. Most of these immune mediators such as chemokines, cytokines and proteases are secreted via two exocytic pathways: constitutive secretion and regulated or “granular” secretion (Lacy and Stow, 2011; Stow et al., 2013). Initially, newly synthesized proteins are transported from the ER to the Golgi complex. In the constitutive pathway these proteins then traffic from the Golgi complex to the cell surface via vesicles and tubulovesicular structures resulting in continuous secretion of cytokines (Stow et al., 2009). Activation of macrophages up regulates exocytosis causing increased cytokine release (Stow et al., 2009). In addition, professional secretory cells like neutrophils or mast cells can secrete proteins through regulated secretion and degranulation (Logan et al., 2003). During regulated secretion immune proteins get sorted from the Golgi complex to specific compartments which include secretory granules (SGs), LROs and secretory lysosomes, where specific stimuli then trigger their release from the cells allowing for a rapid response (Lacy and Stow, 2011). For example, mast cells in response to immunoglobulin E (IgE) receptor ligation contribute to pro-inflammatory responses (Wernersson and Pejler, 2014).

Several Rab GTPases including Rab3, Rab12, Rab27a, and Rab37 have been implicated in the regulation of different steps in secretory pathways associated with immune responses. While all Rab3 isoforms have been linked to exocytosis, Rab3d was the first non-neuronal secretory Rab identified to localize to SGs in mast cells (Tuvim et al., 1999). Rab3d has been implicated in maintaining SG size, however, its role in degranulation remains unclear, as Rab3d-deficient mice do not show changes in regulated exocytosis (Riedel et al., 2002). siRNA mediated knockdown experiments showed that exocytosis by endothelial-cell specific LROs called Weibel-Palade bodies, which are important in angiogenesis, thrombosis and inflammation, required Rab3a, Rab3d, Rab27, and Rab15 (Zografou et al., 2012).

Rab12 is associated with SGs in atrial myocytes (Iida et al., 1996) and implicated in promoting vesicular transport from the cell periphery to the perinuclear region (Iida et al., 2005). The Rab7 effector Rab7-interacting lysosomal protein (RILP) also acts as an effector for Rab12 and it has been suggested that Rab12 counteracts the anterograde transport of SGs along microtubules to inhibit degranulation by acting in between the RILP-dynein complex (Efergan et al., 2016). Moreover, a screening for Rab GTPases that regulates SG exocytosis in mast cells and hence pro-inflammatory responses showed that Rab12 activity is directly regulated in response to immune stimuli (Efergan et al., 2016).

A role for Rab27a in exocytosis and immunity was first demonstrated in Rab27a-deficient mice, which show impaired lytic granule exocytosis (Stinchcombe et al., 2001). Moreover, Rab27a also contributes to the degranulation of neutrophil azurophillic granules (AGs) (for reviews, see references Catz, 2014; Ramadass and Catz, 2016). In addition to direct involvement of Rab27a, its effectors Munc13-4 and JFC1/Slp1 have also been implicated in the secretion of myeloperoxidase from neutrophil AGs (Munafo et al., 2007; Brzezinska et al., 2008; Johnson et al., 2011). Munc14-3 is important for the docking of Rab27a vesicles at the plasma membrane (Johnson et al., 2016). Rab27a-dependent exocytosis has also been implicated in systemic inflammation through secretion of cytokines including tumor necrosis factor-α (TNF-α) (Johnson et al., 2011) and neutrophil infiltration in response to inflammatory stimuli (Johnson et al., 2011; Singh et al., 2012). Rab27a down-regulation correlates with lower neutrophil-mediated tumor cytotoxicity (Bobrie et al., 2012; Yan et al., 2013). In contrast to Rab27a, loss of Rab27b in immune cells only leads to a minor inhibition of AG degranulation (Johnson et al., 2010). Rab27b shares a 71% homology with Rab27a (Fukuda, 2013) but its up-regulation during Rab27a deficiency cannot restore the defect in exocytosis (Johnson et al., 2010). In fact, it seems that Rab27a and Rab27b have opposing effects on mast cell degranulation (Singh et al., 2013). While Rab27b acts as a positive regulator of exocytosis in mast cells, Rab27a acts as a negative regulator of stimulus-dependent exocytosis by modulating SG tethering and docking at the plasma membrane (Mizuno et al., 2007; Singh et al., 2013).

Studies examining insulin exocytosis and TNF-α release by macrophages in response to lipopolysaccharide (LPS) stimulation have suggested the importance of Rab37 in regulated exocytosis (Mori et al., 2011; Ljubicic et al., 2013). Rab37 was originally identified in mast cells (Masuda et al., 2000) and has recently been demonstrated to negatively regulate mast cell granule exocytosis (Higashio et al., 2016). Rab37 can form a complex with Rab27-Munc13-4 on secretory granules and it has been speculated that an effector recruited by Rab37 could be responsible for counteracting the Rab27-Munc13-4-dependent granule secretion (Higashio et al., 2016).

In macrophages, the membrane trafficking pathways that control phagocytosis and cytokine secretion are interconnected. In this way, Rab11 positive recycling endosomes provide membrane for the internalization of pathogens at the phagocytic cup as well as secreting during this process the pro-inflammatory cytokine TNF-α (Murray et al., 2005).

### Autophagy

The cellular degradative pathway of autophagy plays a crucial role in regulating different aspects of the innate and adaptive immunity as well as inflammation. During autophagy macromolecules, organelles or invading microorganisms can be sequestered in a double membrane structure, the autophagosome, which fuses with lysosomes to enable the degradation of its contents (Mizushima, 2007). Several Rab GTPases are involved in the regulation of autophagy, among which Rab7 is the best characterized. Rab7 is recruited to autophagosomes where it regulates the fusion with lysosomes (Gutierrez et al., 2004). Other Rab GTPases implicated at different stages of autophagy are Rab1, Rab5, Rab4, Rab8, Rab9, Rab11, Rab24, Rab32, and Rab33 (Ao et al., 2014; Szatmari and Sass, 2014).

Rab GTPases are involved in the formation of autophagosomes around invading bacteria, as well as in their trafficking to lysosomes for degradation. However, intracellular bacteria have evolved different strategies to avoid autophagosomal targeting by interfering with different Rab GTPases (Huang and Brumell, 2014). Virulent *M. tuberculosis* prevents the accumulation of Rab7 on autophagosomes, thus reducing autophagosome-lysosome fusion and increasing mycobacterial replication (Chandra et al., 2015; Hu et al., 2015). Furthermore, due to its effects on TBK-1 (TANK binding kinase-1) dependent autophagy flux, knockdown of Rab8b in RAW264.7 macrophages leads to increased replication of *M. bovis* BCG after induction of autophagy by starvation (Pilli et al., 2012). The Golgi complex resident Rab30 has been shown to be involved in the targeting of Group A *Streptococcus* (GAS) to autophagosomes to restrict their replication (Oda et al., 2016). While Rab30 knockdown does not affect the recruitment of autophagic adaptor proteins NDP52 and p62, it decreases the association of LC3 to GAS containing autophagosome like vacuoles (Oda et al., 2016). Rab32, which is important for the formation of autophagosomes (Hirota and Tanaka, 2009; Wang et al., 2012), restricts the intracellular survival of *S. typhi* in mouse macrophages (Spano and Galan, 2012). On the other hand, the broad-host range adapted *S. typhimurium* secrets the effectors SopD2 and GtgE, which act as a GTPase activating protein (GAP) and a protease, respectively and promote evasion of Rab32-dependent host immune responses, favoring bacterial survival (Spano, 2016; Spano et al., 2016). This small GTPase is also required for the restriction of intracellular *Listeria* replication (Li et al., 2016). However, the specific role of Rab32-dependent autophagy in these antimicrobial responses remains unknown. The manipulation of the Rab GTPase network by microbes is not only restricted to bacterial pathogens and viruses can also interfere with Rab GTPases implicated in the autophagic pathway. For example, Hepatitis B virus (HBV) activates Rab7 through the action of the precore protein HBe increasing the degradation of virions (Inoue et al., 2015).

## Rab GTPases in adaptive immunity

Innate immune responses induced by pathogen recognition instruct subsequent long lasting adaptive immunity mediated by antigen responsive B and T lymphocytes (Iwasaki and Medzhitov, 2015). To initiate cytotoxic immune responses, T cells must be “activated” by the process of antigen presentation. In antigen presenting cells (APC), pathogens or dead cells internalized by phagocytosis and macropinocytosis are first degraded in phagosomes and endosomes, and subsequently degraded antigens coupled to specific membrane receptors are transported by vesicles to the cell surface (Iwasaki and Medzhitov, 2015). It is therefore not surprising that Rab GTPases play a crucial role during antigen presentation and T cell mediated immunity in APC such as DCs and macrophages (Trombetta and Mellman, 2005). In order to mount an effective T cell response, it is important that antigen processing does not lead to antigen degradation. Therefore, as a strategy to reduce antigen degradation and to drive adaptive responses, Rab GTPase-dependent trafficking contributes to slower acidification and phagosome maturation in DCs (Savina and Amigorena, 2007). Rab27a-dependent trafficking of LROs causes the recruitment of the NADPH oxidase subunit NOX2 to phagosomes (Jancic et al., 2007), where it contributes to slower antigen processing by increasing phagosomal pH (Savina et al., 2006) and reducing phagosomal proteolysis by affecting cathepsins (Rybicka et al., 2012). Rab34 interacts with RILP to regulate lysosomal positioning and fusion with phagosomes (Cantalupo et al., 2001; Wang and Hong, 2002; Kasmapour et al., 2012). Toll-like receptor 4 (TLR4) engagement on DCs in response to LPS stimulation causes Rab34-dependent lysosomal clustering thereby delaying phagosomal maturation and antigen degradation and allowing for better T cell priming (Alloatti et al., 2015).

Phagosomes containing microbial components that engage TLR signaling recruit major histocompatibility complex (MHC) class I molecules from Rab11 positive endosomal recycling compartments (ERC) (Adiko et al., 2015). Rab11 contributes to antigen cross-presentation by trafficking and maintaining MHC class I molecules at the ERC (Nair-Gupta et al., 2014). Additionally, Rab11a has been shown to recruit TLR4 from ERC to bacteria-containing phagosomes, contributing to interferon regulatory factor-3 signaling and IFN-β production, further supporting its role in immune signaling (Husebye et al., 2010). Rab8a, Rab10, Rab7b are among the other Rab GTPases that can modulate TLR4-dependent immune responses (Wang et al., 2007, 2010; Luo et al., 2014).

While not directly implicated in antigen presentation by APCs, Rab9 was recently shown to mediate mitochondrial antigen presentation (Matheoud et al., 2016). In addition to their role in signaling and metabolic functions, mitochondria are important in immune responses and several PAMP-dependent signaling pathways require mitochondria-derived reactive oxygen species (for a comprehensive review, see reference Weinberg et al., 2015). Even though mitochondria are implicated in autoimmunity, the mechanisms responsible for recognition of self-antigens to develop immune tolerance are not well known (Weinberg et al., 2015). Damaged mitochondria are eliminated by mitophagy that in turn limits the presentation of mitochondrial antigens. Recent work has shown the presence of mitochondria derived vesicles (MDVs), which serve as a cellular quality control mechanism whereby damaged mitochondrial components like lipids and outer membrane can be delivered to peroxisomes for degradation (Neuspiel et al., 2008). These MDVs are important for mitochondrial antigen presentation and require Rab9 for their formation, while their fusion with lysosomes occurs in a Rab7-dependent manner (Matheoud et al., 2016).

## Rab GTPase expression in immunity and inflammation

Several studies on immune cells have provided evidence for the transcriptional control of intracellular membrane trafficking proteins. Collectively, these studies have demonstrated the role of immune modulators and microbes in regulating Rab protein expression (Pei et al., 2012). IFN-γ produced by natural killer and natural killer T cells is critical for immunity against viral and bacterial infections and contributes to macrophage activation by increasing phagocytosis and production of pro-inflammatory cytokines (Schoenborn and Wilson, 2007). Macrophages stimulated with IFN-γ show an increase in the expression of Rab5a and Rab20, both of which are important in phagosome maturation (Alvarez-Dominguez and Stahl, 1998; Vieira et al., 2003; Pei et al., 2014, 2015). Furthermore, the expression levels of Rab20 and Rab34 are up-regulated in response to *M. avium* and *M. smegmatis* infection, with Rab10 also up-regulated by *M. smegmatis* infection (Gutierrez et al., 2008).

Given the critical role of Rab5 in controlling both phagosome-early endosome fusion and the maturation of phagosomes into degradative compartments, cytokine-dependent regulation of Rab5 levels could provide control over phagosomal maturation. Along with upregulation through IFN-γ stimulation (Alvarez-Dominguez and Stahl, 1998), Rab5 expression can be up-regulated by interleukins (IL) 4 and 6. IL-4 induces alternate activation of macrophages and together with prostaglandin E2 (PGE2) induces Rab5a expression (Wainszelbaum et al., 2006). IL-4 stimulation of macrophages also results in prolonged retention of Rab5 on phagosomes and a delay in phagosome acidification in a phosphoinositide 3-kinase (PI3K)-dependent manner (Keijzer et al., 2011). Interestingly, IL-4/PGE2 enhance proteolytic activity in phagosomes (Balce et al., 2011). In contrast to IL-4 stimulation, overexpression of Rab5a in macrophages results in enhanced maturation of *Listeria* containing phagosomes (Alvarez-Dominguez and Stahl, 1999). The pro-inflammatory cytokine IL-6 similarly up-regulates Rab5 expression via activation of extracellular signal-regulated kinase (ERK) resulting in an increased fusion of early endosomes and phagosomes (Bhattacharya et al., 2006). Furthermore, the induction of Rab5a increases early endosome homotypic fusion thereby generating enlarged endosomal compartments (Wainszelbaum et al., 2006). The different effects in Rab5 expression after cytokine stimulation when compared with overexpression of Rab5 fusion proteins on phagosome maturation warrant further investigation. It also remains to be determined whether the observed differences in phagosomal acidification and proteolytic activity are linked to changes in Rab5a expression and endosomal morphology.

In contrast, expression of the late endosomal Rab7 is induced by IL-12 in a p38/MAPK-dependent manner (Bhattacharya et al., 2006). Increased Rab7 levels enhance transport of phagosomal cargo to lysosomes and inhibit the survival of intracellular *Salmonella* (Bhattacharya et al., 2006). In addition, the peptidoglycan constituent muramyl dipeptide, which is present in both gram positive and gram negative bacteria, has opposing effects on Rab5 and Rab7 levels in macrophages. Muramyl dipeptide decreases Rab5 levels while increasing Rab7 expression, corresponding with delivery of *Salmonella* to lysosomal compartments (Mukherjee et al., 2002).

Rab20 is an IFN-γ responsive Rab GTPase implicated in phagosome maturation and macropinocytosis (Egami and Araki, 2012a; Pei et al., 2014, 2015). IFN-γ was shown to up-regulate both total Rab20 levels (Pei et al., 2015) and its association with isolated bead-containing phagosomes (Trost et al., 2009). Further supporting the role of Rab20 in immunity, analysis of gene expression in lungs in the mouse model of asthma showed an increase of Rab20 expression after injection with ovalbumin (Malik et al., 2008). Furthermore, microarray analyses revealed Rab20 expression also increases in response to infections with pathogens including *Aspergillus fumigatus* (Cortez et al., 2006), *Streptococcus pyogenes* (Goldmann et al., 2007), and *Listeria monocytogenes* (Tchatalbachev et al., 2010). In addition, microarray data identified an increase in Rab20 levels during mycobacterial infection, which was dependent on NF-KB (Gutierrez et al., 2008). Simultaneous transcriptional profiling of *M. tuberculosis* and its infected host cells by microarrays showed a significant up-regulation of Rab20 in human macrophages but not in DCs (Tailleux et al., 2008). Remarkably, in a recent study of genes associated with the host transcriptional signature in active tuberculosis, Rab20 was the only small GTPase of the Rab family found in this set of 393 genes (Berry et al., 2010).

Supporting the idea that Rab20, together with Rab32, are part of a group of small GTPases linked to inflammation, the up-regulation of both Rab20 and Rab32 during the acute phase of LPS-induced brain inflammation has been reported (Liang et al., 2012). However, the mechanisms or consequences of this up-regulation are not known. Furthermore, high throughput siRNA screening to identify host pathways during *L. monocytogenes* infection in HeLa cells demonstrated that both Rab20 and Rab32 are required for the control of *Listeria* infection (Kuhbacher et al., 2015). While the direct transcriptional regulation of Rab32 in response to cytokines is unclear, some reports have directly linked Rab32 to immune responses in animal models of infection (Liang et al., 2012; Solano-Collado et al., 2016).

## Rab GTPases-associated immune disorders

Given the critical roles of Rab proteins in immune related processes, they have been implicated in several immune disorders. Indeed, multiple genome-wide studies suggest that Rab protein-dependent cellular trafficking events are involved in immune responses (Pei et al., 2012). Disorders associated with dysfunctional Rab GTPase pathways can result from direct dysfunction of Rab proteins or indirectly as a result of defective Rab protein regulators or effectors, and can be genetic or occur during infection due to pathogen-driven processes (for reviews, see references Mitra et al., 2011; Seixas et al., 2013).

Several of the inherited Rab-associated disorders affect LROs and Rab27, Rab38, and Rab32 have been implicated in diseases with underlying defects in LRO trafficking. Interestingly, Rab27a is the only Rab GTPase that is clearly associated with an immune dysfunction in humans. Defects in Rab27a-dependent trafficking of melanosomes in melanocytes that affects the transfer of melanin to keratinocytes are associated with Griscelli syndrome, characterized by hair and skin hypopigmentation and impaired secretion of lytic granules from cytotoxic T lymphocytes, leading to immune-deficiencies (Menasche et al., 2000). Mutations in the Rab escort protein 1 (REP1), which is essential for prenylation of Rab GTPases, disrupt Rab27a trafficking through accumulation of unprenylated Rab27a, causing choroideremia (van den Hurk et al., 1997; Mitra et al., 2011). Moreover, defects in Rab27a-dependent transport of melanosomes in retinal-pigment epithelium is speculated to contribute to its degeneration leading to the loss of peripheral vision and night blindness associated with the disease (Mitra et al., 2011). Mutations in the Rab GTPase Rab38 also result in defective LRO trafficking and have been implicated in Hermansky–Pudlak syndrome in *chocolate* mice (Loftus et al., 2002). This hypopigmentation disorder is associated with impaired clotting due to the absence of platelet dense granules (Huizing et al., 2000). Rab38 and its close homolog Rab32 were also reported to control *Salmonella* and *Listeria* infection (Spano and Galan, 2012; Li et al., 2016). Furthermore, genetic evidence supports a role for Rab32 in controlling leprosy, caused by *M. leprae* (Zhang et al., 2011). While, the best-characterized function of Rab32 is its role in LRO trafficking, whether the Rab32-dependent LRO trafficking contributes to phagolysosome formation and pathogen clearance remains unknown.

Aberrant Rab protein expression is associated with diseases where chronic inflammation is speculated to contribute to disease causation and progression, including several types of cancers (Chia and Tang, 2009). Several hallmarks of cancer cells, such as altered cell polarity, require dysfunction of membrane trafficking events regulated by Rab GTPases. While the direct involvement of Rab proteins in cancer is not well established, abnormal expression of various Rab GTPases has been detected in several cancers (Chia and Tang, 2009; Goldenring, 2013), whereby aberrant Rab expression can be linked to varying phenotypes in different cancers. Rab25 and Rab21, which are involved in the trafficking of integrin receptors, are for example up-regulated in ovarian cancers and potentially promote cancer cell invasion (Cheng et al., 2004; Pellinen et al., 2006; Caswell et al., 2007). On the other hand, loss of Rab25 was associated with triple-negative breast cancer and head and neck cancers (Goldenring, 2013).

In addition to aberrant Rab expression, mistargeting of Rab GTPases or changes in their activity and localisation by posttranslational modifications is associated with various disorders. Chron's disease (CD) is a type of inflammatory bowel disease (IBD) characterized by a chronic inflammation of the gastrointestinal tract (Landy et al., 2016). This disease is associated with defects in cell-cell junctions resulting in loss of mucosal barrier integrity and increased permeability of the intestine (Teshima et al., 2012). Rab13 regulates the structure and function of tight junctions (Marzesco et al., 2002) and the mistargeting of Rab13 to basolateral sites observed in CD patients (Ohira et al., 2009) suggests a contribution of Rab13 dysfunction in CD. Several neurodegenerative disorders, including Parkinson's disease (PD), are associated with inflammation, where it is believed to cause neuronal degeneration and contribute to disease progression (Russo et al., 2014). Multiple studies support the role of leucine-rich repeat kinase 2 (LRRK2) in microglia-mediated inflammatory responses through regulation of vesicle trafficking, endocytosis and secretion (Russo et al., 2014). Interestingly, a recent phosphoproteomic screening revealed that several Rab GTPases act as LRRK2 substrates (Steger et al., 2016). This indicates a potential role for LRRK2-dependent Rab phosphorylation in regulating vesicle trafficking implicated in PD associated neuroinflammation.

## Conclusions

In the last few years it has become evident that Rab GTPases, by regulating fundamental cellular processes, also regulate many important aspects of immune responses. Whereas some processes such as phagosome maturation and antigen presentation are well described, it is often not considered that secretion of cytokines relies on exocytosis; a membrane trafficking pathway regulated by Rab GTPases among other molecules. In addition, the expression of Rab GTPases is heavily regulated by cytokines, and there is a network of Rab proteins linked to various inflammatory processes that include Rab20 and Rab32. Future work will define the molecular basis of Rab gene expression during disease and their role during infection and immunity. *In vivo* studies with knock-out mice have demonstrated antimicrobial roles for Rab20 (Schnettger et al., 2017) and Rab32 (Solano-Collado et al., 2016), however roles of other Rab proteins using *in vivo* models remain largely undefined. Extending findings from *in vitro* studies to *in vivo* models will be crucial in understanding the roles of other Rab proteins in immune responses.

## Author contributions

All authors listed have made a substantial, direct and intellectual contribution to the work, and approved it for publication.

### Conflict of interest statement

The authors declare that the research was conducted in the absence of any commercial or financial relationships that could be construed as a potential conflict of interest.
